# Endoscopic resection of a large distal common bile duct adenoma with focal malignancy: palliative approach in a high-risk patient

**DOI:** 10.1055/a-2598-5130

**Published:** 2025-05-28

**Authors:** Yuyan Zhang, Miao Zhang, Yi Mou, Pan Li, Bing Hu

**Affiliations:** 1 Department of Gastroenterology and Hepatology, West China Hospital, Sichuan University, Chengdu, China; 2 Department of Gastroenterology and Hepatology, The First Affiliated Hospital of Chongqing Medical University, Chongqing, China

**Fig. 1:**
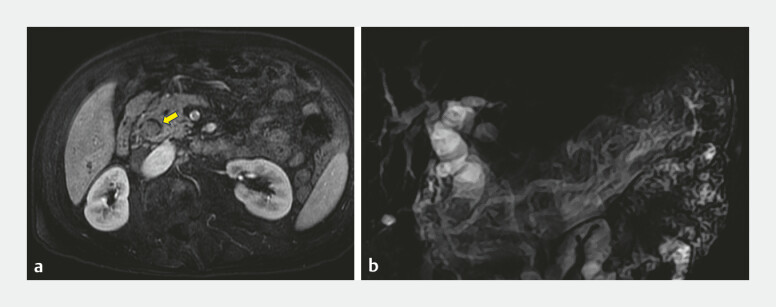
Magnetic resonance cholangiopancreatography: **a** A soft-tissue nodule (yellow arrow) measuring 1.9 × 1.4 cm in the distal CBD, **b** The upstream bile duct was significantly enlarged. Abbreviation: CBD, common bile duct.

**Fig. 2:**
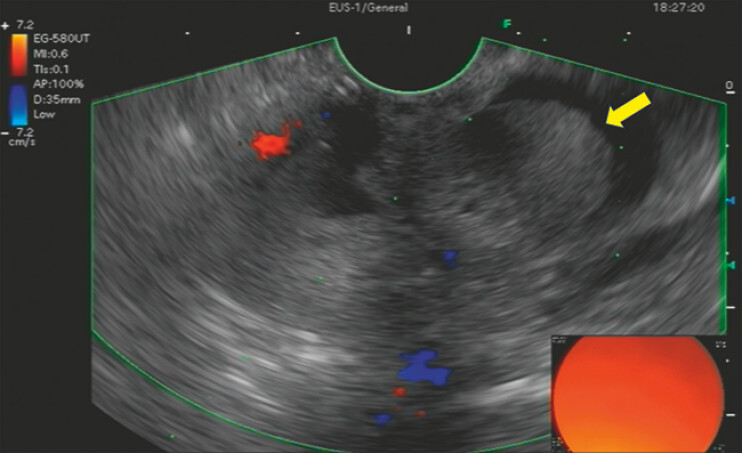
Endoscopic ultrasonography demonstrated an oval and non-shadowing polypoid mass (yellow arrow) in the distal CBD. Abbreviation: CBD, common bile duct.

**Fig. 3:**
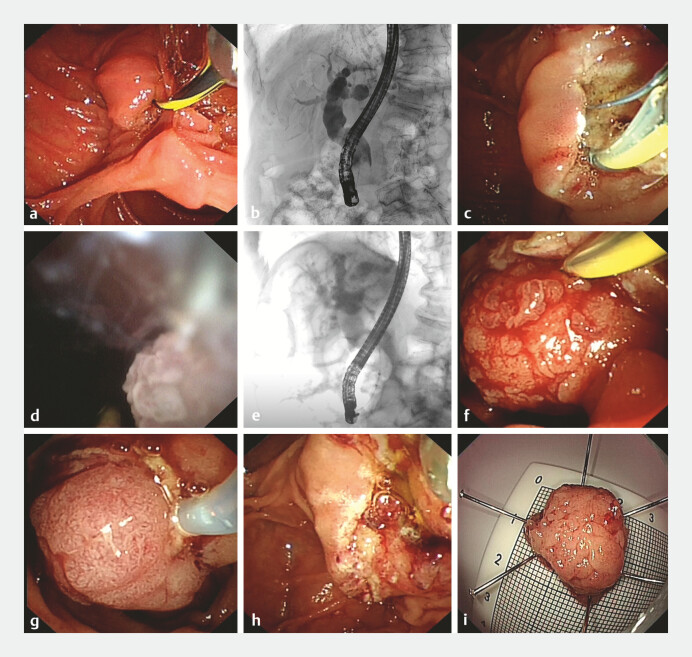
Endoscopic retrograde cholangiopancreatography and resection procedures: **a** The duodenal papilla is significantly enlarged, with normal surface mucosa; **b** Cholangiogram showing an irregular-shaped filling defect in the distal CBD, measuring about 2.5 cm in length, immediately proximal to the duodenal papilla; **c** Endoscopic sphincterotomy was performed, creating a 0.8 cm incision; **d** Transoral choledochoscopy discovered a villous neoplasm in the distal CBD; **e, f** Fluoroscopic and endoscopic view of the lesion being dragged out through balloon extraction; **g** This lesion was resected using hot snare polypectomy; **h** After resection, neither bleeding nor perforation was observed; **i** The specimen was retrieved using a basket and measured approximately 2.2 × 2.2cm. Abbreviation: CBD, common bile duct.

A 79-year-old man presented with 3 months of bloating/anorexia and 20 days of fever. Magnetic resonance cholangiopancreatography revealed a 1.9 × 1.4 cm nodule in the distal common bile duct (CBD) near the duodenal papilla, accompanied by upstream biliary dilation ( Fig. 1 ). Endoscopic ultrasonography identified a polypoid mass suspicious of adenoma ( Fig. 2 ). The patient was considered a poor surgical candidate due to his advanced age and multiple comorbidities (chronic bronchitis, emphysema, and anemia). The endoscopic intervention was pursued. Endoscopic retrograde cholangiopancreatography revealed an enlarged duodenal papilla with intact mucosa ( Fig. 3 **a** ) and an irregular filling defect in the distal CBD, proximal to the duodenal papilla, with markedly dilated upstream bile ducts ( Fig. 3 **b** ). Endoscopic sphincterotomy was performed, creating a 0.8 cm incision ( Fig. 3 **c** ). Then, a choledochoscope was advanced into the CBD, identifying a spherical villous neoplasm with intact villi ( Fig. 3 **d** ). Under fluoroscopic guidance, the lesion was extracted into the duodenal lumen using a balloon ( Fig. 3 **e, f** ), excised via hot snare polypectomy ( Fig. 3 **g** ), and retrieved with a basket. The resected lesion measured approximately 2.2 × 2.2 cm ( Fig. 3 **i** ). No bleeding or perforation was observed ( Fig. 3 **h** ), and bile flow was restored ( Video 1 ).

Palliative endoscopic resection of a large adenoma with focal malignancy in the distal common bile duct.Video 1

The 40-minute procedure was uneventful. Histopathology confirmed villous adenoma with focal high-grade dysplasia and carcinoma involving the lamina propria, though margins were unable to be evaluated. The patient’s symptoms improved, and at the 6-month follow-up, no discomfort was reported, but he declined further surveillance due to socioeconomic factors.

We present endoscopic resection of a large focal malignant adenoma in the distal CBD as a palliative approach, which restored biliary tract patency, alleviated symptoms, achieved cytoreduction, and enabled definitive diagnosis. Endoscopic therapy may serve as a viable alternative for select patients with distal CBD polypoid lesions, particularly elderly individuals with prohibitive surgical risk.

Endoscopy_UCTN_Code_TTT_1AR_2AF

